# Sensation Novels

**Published:** 1863-07

**Authors:** 


### Art. VII.—SENSATION NOVELS.

The new school of fiction, commonly described by the title of the present article, is in many respects a phenomenon too remarkable to be left wholly without notice in our pages. Many years ago, when the author of *Oliver Twist* revealed to the patrons of circulating libraries the arcana of thievery and prostitution, it was the custom to remark that these evils were too great to be discussed in any but a serious spirit. They should, it was said, be examined and dealt with by statesmen and philanthropists, but be excluded from the domain of polite literature. Fagin and the Dodger, Bill Sykes and Nancy, were admirable subjects for legislation, or for the exertions of town missionaries, but were hardly calculated to elevate the minds of young ladies, or to give a desirable tone to drawing-room conversation. Since then, however, the progress of time has wrought great changes, and our light literature, first becoming "fast," has latterly developed itself into something positively vicious. A heroine who was not an adulteress and a poisoner would disgust a modern novel-reader, and would prevent him from following, even as far as the second volume, the fortunes of a person so uninteresting. It is worth while, we think, briefly to inquire into the causes that have worked so great a revolution.

Among possible explanations of the problem, a prominent place may be assigned to feebleness of writing, and want of inventive power on the part of authors, leading them to supply deficient interest by horrible and startling incidents, or by the introduction of characters that appeal to a morbid and prurient curiosity. This explanation, however, does not in any way apply to masters of the art, or, as a rule, to the originators of the "sensation novel" movement; and although it may meet the case of imitators, and of writers more or less weak, it leaves unsolved the question of the sources of the interest felt, now-a-days, in the crimes or immoralities of persons who, by reason of beauty, or wealth, or fashion, or any other social distinction, are a little conspicuous above their surrounding circles. The interest is, we think, in the crimes absolutely, not in their counterfeit presentments in the pages of a novel; and it attaches itself in a far greater degree, therefore, to the actual than to the fictitious. Writers have not been slow to perceive that the columns of the daily papers were becoming formidable rivals to quiet novels; and it is probably only a result of the admirable organization of the literary market, that a supply of acceptable fiction has so closely followed, or has even in some degree anticipated and created the demand. If this be so, and if a popular craving for excitement is more fully satisfied now than at any former time, only on account of the facilities afforded by competing publishers, and cheap paper, and Mr. Mudie, and innumerable local libraries, and by an extension of education and wealth that has increased fifty-fold the possible writers and the certain devourers of fiction, it follows that the craving for excitement itself is the only element in the matter that presents much interest to the psychologist, or requires much examination at his hands. Is this craving then in any sense a novelty, either in its essential nature, or in its direction, or in its extent? and if so, of what nature are the evils it produces, and where are we to seek their remedy.

In reply to these questions, it appears to us that the love of excitement incidental to the idle members of prosperous communities is showing itself, to some degree, in a direction new to the present generation, and in an extent which, although vastly exceeding anything ever before witnessed, is only commensurate with the immense increase and wonderful diffusion of wealth and superficial knowledge. People with nothing to do, and with sufficient money to live in luxury, have always had, and from the nature of the human mind always must have, a strong desire for "sensations"—a desire that has invariably found gratification in the acts and sayings of conspicuous criminals. When ladies and gentlemen escorted renowned highwaymen to Tyburn, and when gambling and drinking were the universal customs of polite society, this desire was at least as glaringly shown as it is in the present day, even although it did not extend so far, or spread so deeply, on account of the different social conditions of the time. A period followed in which the public craving for excitement was satisfied, to a very great extent, by a bloody, protracted, and adventurous war; a war so protracted as seriously to affect the growth and increase of the national prosperity, and to diminish the numbers of the idle and luxurious classes. Peace brought with it a long arrear of urgently-needed political reforms; to be met by a community impoverished and burdened by debt, and agitated by all the stormy passions that political reforms excite. It was not until the national acceptance of Free Trade had brought our institutions nearly parallel with the intelligence of the time, that we entered upon that course of prosperity in which the war with Russia, the Indian mutiny, the Lancashire distress, have been but as passing clouds upon an April day of sunshine. Our traders and manufacturers, collectively growing rich with unexampled rapidity, have, by their families, enormously swelled the number of the wealthy unemployed, to whom the craving for sensation comes with a force intensified by the absence, or by the general condemnation, of many of the most stimulating resources of an earlier period. The pattern set by an exemplary court and a decorous nobility leading to the almost universal abandonment of gambling and drinking, has added to the natural consequences of those vices the absolute demoralisation that attends upon practices held in universal disrepute, and has left them as the uninvaded and unenvied privileges of some of the vilest of mankind. For well-conducted idlers, either male or female, there was no alternative but to join the Count of Toulouse in his melancholy chant:—

> Oh dear, what will become of us ?
> Oh dear, what shall we do ?
> We shall die of blue devils if some of us
> Can't find out something that's new.

For a certain time the want of sensation impressed itself upon literature and the stage, and the hero of *Used Up* was but a caricature of a phenomenon far too real and too frequent. The males thus suffering have found resources in muscular Christianity and the rifle movement; the females, in the fabrication and perusal of sensation literature.

We have said already that the craving for excitement has taken a direction in one respect new to the present generation. We refer to the interest excited by sexual immorality.

It seems at first sight not unreasonable to believe that this interest marks a decline in the public standard of right in such matters, and shows that playgoers and novel readers, led on by easy stages from the *Lady with the Camellias* to *Lady Audley*, are undergoing that gradual process of hardening, sympathy, and attraction, described in the familiar line—

"We first endure, then pity, then embrace."

We have even heard it hinted, nay, positively maintained, that the decline of female (and consequently also of male) virtue, in the present generation, has been a fact too remarkable to escape even the least careful observer, and that it has been chiefly due to no less a cause than *crinoline*.

As a ground for this opinion, it has been stated that the present style of female dress is utterly destructive to modesty, because it exposes the person in a very great degree, and yet in a degree short of that Spartan nudity that tends to repress inordinate desires. Every woman who walks up a hill, or down a hill, who stoops forward, who turns quickly round, who gets over a stile, who enters a railway carriage, who joins in rotatory dances, or who ascends or descends a staircase, exhibits to observers favourably placed a great deal that is commonly supposed to remain and ought to be concealed. Among the many Crimean stories of that brave and skilful officer, the late Admiral Boxer, it was related that, in Balaklava harbour, his boat passed under a transport just as the wife of a titled and distinguished military commander was about to descend the ship's side. "I say, ma'am," roared the Admiral, "when you've been at sea as long as I have, you'll wear trousers!" So lately as last summer, on the occasion of a picnic in an undulating locality, we saw the utmost consternation excited by a lady who, having great need of the warning voice of the Admiral, nevertheless roamed about the slopes in quest of wild strawberries, stooping, every now and then, to secure some fragrant and tempting prize. Not to multiply instances, we may simply put it as a well-known fact that all women, now-a-days, do habitually display to the public gaze either their trousers, or the parts that trousers were designed to cover. Every woman sees other women exhibit their legs, and every woman, therefore, knows that she exhibits her own. The rising generation of young women and girls have been trained to this display from their infancy. The result, it is argued, is the utter destruction of that modesty which is one of the strongest outposts of virtue. Modesty being destroyed, chastity becomes a mere matter of prudence and caution; and girls and women who are restrained by the fear of consequences from giving the rein to their own passions, still find a fictitious excitement in reading about, and imagining, the gratified passions of others. For this purpose an euphuistic language is required, in which the trulls and strumpets of homely English are known as "soiled doves" and "pretty horsebreakers," and become, under the influence of this most modern example of "the terrible imposture and force of words," subjects of conversation for women called virtuous, who still adopt their phraseology, imitate their dress, and endeavour to imagine their delights.

In all this there is, we fear, some considerable admixture of truth; and yet, we hope a good deal of error also. It is difficult to speculate on the moral influence of an almost universal custom. If crinoline wearers were as scarce as bloomers, there can be little doubt that an exhibition of legs, thus exceptional, would have a strong tendency to produce many of the effects above described. But, save for a few "strong-minded females," in no respect, certainly, to be taken as examples to their sex, all women wear crinoline. All women, virtuous as well as vicious, display their legs in almost equal proportions; and it follows that many must do so without sustaining any hurt. In Turkey, where concealment of the face is the point of decorum, we were once pedestrianizing in a region little visited by Giaours. At a little hamlet half a dozen peasant women, naked to the waist and unveiled, were winnowing corn. Our approach and request for some water to drink, elicited a perfect tempest of howls and yells; the hags covering their hideous faces with their grimy fingers, and shouting, Go, go! As usual pertinacity carried the day; and one of the women went to her house to obtain the object of desire. In a few moments she returned, easy and unembarrassed, her features shrouded behind a fragment of dirty rag, her hands carrying the wished-for water, but with no other addition to her clothing. With Oriental courtesy and politeness she gave us to drink and wished us good speed upon our journey, happy in the consciousness that she was veiled, and her face concealed from the gaze of man. Doubtless she would regard the European exposure of the features as a probable cause of all the evils that some Europeans anticipate from exposure of the legs. And so, after all, custom becomes a great power in such matters; and that which is customary ceases to be indecent, or to produce the effects of indecency. If crinoline should continue to be the *mode*, we may fairly hope that female modesty, even if at first somewhat startled, will eventually resume its sway.

There is, moreover, some ground to believe that the interest felt by the educated classes in sexual immorality increases in proportion to the increasing variety of the offence. There have been times, not very remote, in English history, when immorality was uninteresting and prosaic, simply by reason of its universality. In the present day, ladies of station who offend, place themselves by a single effort in the position of distinguished criminals, and excite a share in the concern felt about the conduct and welfare of such persons as Mr. Leopold Redpath or the late Mrs. Manning. We see no reason to fear that this concern will in the least degree tend to increase the crimes of swindling or homicide; and we do not fear either, that the eager perusal of the unsavoury revelations made before Sir Cresswell Cresswell will, as a rule, in any appreciable degree undermine the virtue of our wives and daughters. Perhaps the ladies may learn many things about which they would have done better to remain in ignorance. But among other things they will at least learn the unerring action of the Nemesis that waits upon sin, the certainty of the punishment that follows, it may be, *pede claudo*, but that still follows the transgression of the moral law:—

> "Though the mills of God grind slowly, yet they grind
> exceeding small,
> Though with patience He stands waiting, with exactness
> grinds He all."

And if such be the lesson taught by the current realities of life around us, such will also, in the main, be the lesson taught by the fiction that reflects that life, and that must reflect it faithfully, in order to attract and retain an audience. Let us take such an example as *Aurora Floyd*. Early in life, the heroine has a girl's fancy for a handsome animal. There is no more familiar fact than the broad difference between such a fancy and true feminine affection. The fancy may lead to an affection, if it be in the first place for a worthy object, and if it be indulged and acted upon. If not acted upon, it will die a natural death from exhaustion, from "old age and natural decay," speedy in exact proportion to its violence. If acted upon, and the object of it prove unworthy or distasteful, it will be likely to change into intense repugnance. Based upon this foundation, the whole novel may be regarded as an admonition to young ladies not to let their early fancies run away with them, on pain of suffering great misery and annoyance. And although a sensation novelist must step a little over the bounds of probability, although clandestine marriages with grooms are unfrequent, and although, when contracted, they usually involve a totally different chain of consequences from those imagined by Miss Braddon—still, young ladies who read newspapers will not, on the whole, learn much previously unknown evil from the romance; and they will be furnished with an additional incentive to the exercise of caution and prudence with regard to the degree in which their fancies are to be indulged. The world, it is trite to say, moves fast, evil of every sort is rampant and unconcealed around us; and it is possible that "Sensation literature" may become a substitute, not altogether to be despised, for the didactic teaching that was in vogue with an earlier generation.

---

